# Political rights of persons with disability in the Zimbabwean media

**DOI:** 10.4102/ajod.v14i0.1596

**Published:** 2025-06-30

**Authors:** Priccilar Vengesai

**Affiliations:** 1 Department of Jurisprudence, College of Law, University of South Africa, Pretoria, South Africa

**Keywords:** political rights, media, Zimbabwe, disability, visual impairment, hearing impairment

## Abstract

**Background:**

Political rights are crucial for all individuals, especially marginalised groups such as people with disabilities (PWDs). The Constitution of Zimbabwe specifically reserves two seats in the Senate for PWDs. While this is commendable, the current political climate in Zimbabwe does not sufficiently address PWDs’ political inclusion, necessitating further action.

**Objectives:**

This study aimed to firstly describe diverging definitions of disability and highlight that persons with visual and hearing impairments are excluded from the media, thus affecting their political engagement. Secondly, to build upon this assertion and elucidate the necessity of enhancing media access for PWDs to improve their political engagement.

**Method:**

A qualitative document-based methodology was utilised.

**Results:**

People with visual and hearing impairments face considerable barriers in accessing media content during and following electoral processes, effectively constraining their political participation.

**Conclusion:**

Political parties should ensure the inclusion of sign language interpreters during election campaigns and provide their manifestos in Braille to enhance media accessibility by people with visual and hearing impairments. Additionally, the integration of sign language and Braille into educational curricula may foster more effective political engagement through various media channels for PWDs. Furthermore, training journalists in sign language and Braille may improve their communication for people with visual and hearing impairments.

**Contribution:**

This study reveals significant challenges encountered by PWDs in accessing media, which exposes barriers to their political participation. To address these obstacles to accessing media, practical solutions are proposed that may improve the representation of PWDs in political roles.

## Introduction

In this contemporary era, a democratic society is predicated on the principle that all citizens, including marginalised demographics, should be allowed to engage in the political process. According to Maphosa, Moyo and Moyo (2019:113), this means, ‘equal access to the vote, stand for public office and participate in electoral processes as election officials or observers’. It is widely acknowledged that numerous ordinary citizens in Zimbabwe face significant challenges, including resource limitations, political violence, restricted access to media and insufficient educational opportunities during electoral processes. Marginalised groups, particularly people with disabilities (PWDs), have exacerbated difficulties because of additional environmental, attitudinal and institutional barriers that hinder their ability to fully participate in the political arena (Ndhlovu & Mudzingwa 2022:270).

It is broadly recognised that PWDs represent a substantial segment of the global population. Scholarly consensus indicates that the PWDs’ community represents approximately 15% – 16% of the world’s populace, amounting to over one billion people (Kołłątaj et al. 2023:595; Department of Public Service, Labour and Social Welfare 2021:19; Pillay, Saruchera & Chivandire 2023:21). Studies further indicate that over 80% of PWDs reside in developing nations, with women comprising more than half of this demography (Maphosa, Moyo & Moyo 2019:113; Department of Public Service, Labour and Social Welfare 2021:21; Pillay et al. 2023:21). The available survey data estimate that PWDs constitute 7% of the Zimbabwean population (UNICEF Zimbabwe, n.d.). People with disabilities are, therefore, part of Zimbabwe’s diverse population and have an equal right to participate actively in the politics of the day with the rest of the society (Pan & Yaris 2023:147).

Despite constituting a significant proportion of the population, the political representation of PWDs remains disproportionately low (Zimbabwe Electoral Commission 2020:19; Zimbabwe Election Support Network 2018:55; Zimbabwe Situation 2015). In the 2018 Zimbabwean harmonised election, out of 23 presidential candidates, only one candidate, Elton Mangoma, had declared a disability (Zimbabwe Election Support Network 2018:55; Zimbabwe Situation 2015). In the 2023 elections, among the 11 candidates vying for the presidency, there was no representative from PWDs (Herald 2023a). In contrast to the increased representation of previously marginalised groups, such as women in political institutions like parliaments and cabinets, PWDs remain notably absent from the political sphere (Maphosa, Moyo & Moyo 2019:117). The current state of Zimbabwean politics, therefore, highlights a significant imbalance in political representation, with a lack of proportional participation from PWDs.

This status quo contradicts the human rights model of disability, which mandates equal access to human rights, for PWDs, as enjoyed by the broader society (Sedova 2024:2). Numerous scholars focusing on the African perspective (Oluchina 2015) and the Zimbabwean context (Dziva, Shoko & Zvobgo 2018; Maphosa, Moyo & Moyo 2019; Mtetwa 2016; Peta & Moyo 2019) have established that the political participation of PWDs is a fundamental right that must be upheld. This article builds upon this assertion and elucidates the necessity of enhancing media access for PWDs to improve their political engagement. It begins by offering a comprehensive understanding of disability, specifically highlighting that people with visual and hearing impairments represent the most marginalised groups within the media landscape. It then discusses the relationship between access to media and political participation. Access to media by PWDs is addressed in the context of identified sources of media in Zimbabwe. This study also provides recommendations to enhance media access for PWDs.

### Disability concept in context

Scholars and several legal frameworks have suggested varying definitions of disability. Maphosa, Moyo and Moyo (2019:115) pointed out that ‘there is no universally agreed definition of disability’. Peta and Moyo (2019:86) concur with this view as they note that defining disability is very complex because there is no globally accepted definition. Furthermore, deciding on a precise definition of disability has proven to be futile because disability is intricate, multi-faceted, controversial and dynamic (Kołłątaj et al. 2023:595). Accordingly, Mtetwa (2016:32) suggests that when discussing disability issues, it is initially important to clarify terms, hence the discussion of the meaning of disability in this section.

In the Constitution of Zimbabwe (Zimbabwe Government 2013) (the Constitution), disability has been conceptualised in various ways. Section 22(1) of the Constitution refers to PWDs as ‘persons with physical and mental disabilities’, while Section 22(4) employs the term ‘PWDs’. The lack of uniformity in the terminological framework within the Constitution presents a significant challenge, leading to potentially conflicting interpretations of the nation’s supreme law (Mtetwa 2016:32). For example, when interpreting persons with physical and mental disabilities, it excludes sensory disabilities such as visual and hearing disabilities (Mtetwa 2016:32).

The following definition of disability is found in Section 2 of the *Zimbabwean Disabled Persons Act* (Zimbabwe Government 1992):

… a person with a physical, mental or sensory disability, including a visual, hearing or speech functional disability, which gives rise to physical, cultural or social barriers inhibiting him from participating at an equal level with other members of society in activities, undertakings or fields of employment that are open to other members of society. (p. 2)

Although this definition is more detailed than the one in the Constitution, in that it acknowledges social barriers as constituting disabilities, it fails to acknowledge intellectual disabilities.

Given the fluid nature of definitions of disability and considering ongoing social, political and medical advancements (Kołłątaj et al. 2023:595), it is necessary to establish a working definition for this article. To ensure clarity and coherence, this article adopted the definition from the National Disability Policy (Department of Public Service, Labour and Social Welfare 2021:16), which is similar to the one outlined in the Convention on the Rights of Persons with Disabilities (CRPD) (United Nations 2006). Article 1 of the CRPD provides that:

Persons with disabilities include those who have long-term physical, mental, intellectual or sensory impairments which in interaction with various barriers may hinder their full and effective participation in society on an equal basis with others. (p. 4)

This definition is broad enough to include all forms of disabilities. However, common disabilities in Zimbabwe are physical (31%), visual (24%), multiple (13%), hearing (11%), intellectual (8%) and mental (6%) (Department of Public Service, Labour and Social Welfare 2021:20). Despite the diverse range of disabilities present in Zimbabwe, access to media for individuals within this population is inconsistent. This disparity in media access is closely aligned with the specific nature of the impairment, as identified by Virendrakumar et al. (2017:21). Furthermore, the accessibility of different media sources varies, impacting the extent to which PWDs can engage with information and communication platforms.

In Zimbabwe, the media landscape encompasses television, radio, print journalism and social media platforms (Zirima 2020:1). Because of the primary modalities of media, visual and auditory, people with visual and hearing impairments encounter greater barriers in accessing media sources than other forms of disability. According to Naipal and Rampersad (2018:1), ‘visual impairment is a condition of reduced visual performance that cannot be remedied by refractive correction (spectacles or contact lenses), surgery or medical methods’. Hearing impairment is an inability to hear effectively (World Health Organization [WHO] 2024). While people with visual impairments often encounter significant barriers to accessing print media, people with hearing impairments frequently face challenges in engaging with auditory language from media sources (Witsken 2011:774). Consequently, people with visual and hearing impairments disproportionately experience restricted access to various media sources because of the distinctive challenges associated with these disabilities.

### Human rights model approach

The human rights model of disability was applied as the theoretical basis for this article, which necessitates the acknowledgement of the dignity of PWDs by ensuring their equal enjoyment of rights at par with the rest of society (Sedova 2024:2). The human rights model shifts the focus from regarding PWDs as mere recipients of medical or charitable aid to acknowledging them as active agents within their communities (Skarstad 2024:25). It regards them as ‘subjects with rights, capable of claiming those rights making decisions based on free consent and sufficient information and becoming active members of society’ (Pan & Yanis 2023:148). This paradigm shift indicates that PWDs possess the same agency as other members of society. The human rights model is predicated on international human rights frameworks that transcend mere procedural democracy, emphasising the necessity of recognising and upholding the rights of PWDs (Pan & Yanis 2023:148). Consequently, ensuring access to media and political participation is essential for PWDs, as these rights are intrinsic to their dignity and humanity.

## Research methods and design

This study utilised a qualitative document-based research method in gathering data. Through the University of South Africa’s E-resources (online library), several data sources were accessed, which include peer-reviewed journals, electronic books and book chapters. These data sources were accessed from electronic databases, including Web Science, Pro Quest and Science Direct. These data were supplemented through a Google search of specific websites, including the Zimbabwe Electoral Commission, United Nations, World Health Organizations, African Union, prominent Zimbabwean newspapers, Zimbabwe Broadcasting Corporation and Veritaszim. These websites gave me access to online international conventions, case laws, Zimbabwean legislation and the Zimbabwean sources of media.

The sources of data were purposively selected. A systematic analysis was employed to examine the data, focusing on the mechanisms through which individuals with disabilities engage with media to facilitate their political participation. The analysis covered the legal framework governing media from international, regional and Zimbabwean perspectives. Within this context, various media sources were identified in Zimbabwe and drew extracts to exemplify how media were utilised for political engagement during past elections and subsequent periods. Furthermore, the analysis detailed the identified media sources and highlighted the extent to which people with visual and hearing impairments are marginalised in the media discourse.

## Media and political participation

### International and regional spectrum

The evolution of the perception of PWDs was heralded by the CRPD (United nations 2006), which Zimbabwe formally ratified on 23 September 2013. The CRPD’s (United Nations 2006) primary aim is to address the physical and social obstacles encountered by PWDs in enjoying their rights (De Beco 2019:49). The CRPD (United Nations 2006) reframed the understanding of PWDs, shifting the perspective from one of charity to recognising them as holders of participatory human rights. This transition emphasises empowerment, autonomy and the obligation of states to ensure equal opportunities and inclusion in society. This shift transformed ‘PWDs from passive recipients of aid to fully empowered citizens who enjoy equal rights and protection …’ (Virendrakumar et al. 2017:4). These rights include access to media and political participation.

In Article 21 of the CRPD (United nations 2006), it is stated that:

State parties shall take all appropriate measures to ensure that persons with disabilities can exercise the right to freedom of expression and opinion, including the freedom to seek, receive and impart information and ideas on an equal basis with others and through all forms of communication of their choice, as defined in Article 2 of the present Convention. (p. 14)

The CRPD (United Nations 2006) does not only mandate state parties to grant PWDs access to information but also requires them to identify and remove barriers that hinder PWDs from accessing information and communication (Article 9(1)(b)). Additionally, Article 9(2) (f–g) of the CRPD (United Nations 2006) advocates for the facilitation of their access to new information and communication technologies and systems. Article 4(1)(h) of CRPD (United Nations 2006) acknowledges the need for assistance for PWDs to access information, thus placing an obligation on states to provide them with information mobility aids and any other necessary support and assistance for information access. At its core, the CRPD underscores the necessity of accommodating the unique needs of PWDs. This approach ensures that they can fully exercise their human rights at par with other members of society (Degener & Quinn 2002:13).

The United Nations CRPD Committee (2018:para 9) states that the human rights model of disability recognises that human rights are ‘interdependent, interrelated, and indivisible’. Consequently, while access to media constitutes a fundamental human right in itself, it also facilitates the realisation of the right to political participation. Among other rights, the CRPD (United Nations 2006) establishes the fundamental political rights of persons with disabilities, empowering them to assert their rights and actively participate in crucial decision-making processes. In essence, the CRPD (United Nations 2006) strives to provide PWDs with a voice, agency and the means to effect positive change in their communities and societies (De Beco 2019:49).

Relevant to the political rights of PWDs is Article 29 of the CRPD (United Nations 2006), which provides the following:

States Parties shall guarantee to persons with disabilities political rights and the opportunity to enjoy them on an equal basis with others, and shall undertake to:

Ensure that persons with disabilities can effectively and fully participate in political and public life on an equal basis with others, directly or through freely chosen representatives, including the right and opportunity for persons with disabilities to vote and be elected, inter alia, by … (p. 21)

The essence of Article 29 of the CRPD (United Nations 2006) is that political participation for PWDs enables them to voice their views on the governance of their country (Oluchina 2015:311). Political rights are exercised through various means, including voting and standing for political positions (Maphosa, Moyo & Moyo 2019:117; Oluchina 2015:312). Accommodative measures in each respective mode of political participation are required to minimise the barriers experienced by PWDs in their political participation (De Beco 2019:49; Oluchina 2015:313). Media enhances democracy by providing citizens with essential tools for engaging in political processes (Mathe & Osunkunke 2019:1).

In Africa, there is a Protocol to the African Charter on Human and People’s Rights on the Rights of Persons with Disabilities in Africa (Africa Disability Rights Protocol) (African Union 2018), which Zimbabwe ratified as of May 2024. Articles 23 and 24 of the Africa Disability Rights Protocol elucidate the rights of PWDs to free expression and access to information, and there is a requirement for reasonable accommodation measures that are supposed to be taken, especially for people with hearing and visual disabilities. The African Commission on Human and People’s Rights (African Commission) clarified the importance of freedom of expression in political participation in the case of *Media Rights, Constitutional Rights Project v Nigeria*, (1996) which was adjudicated under the Freedom of Expression Right in Article 9 of the African Charter on Human and People’s Rights (ACHPR) (African Union 1981). In this case, the African Commission determined that the Nigerian Government’s prohibition of two magazines constituted an infringement of Article 9 of the ACHPR (African Union 1981). The African Commission in reaching this decision held that, concerning Article 9 *Media Rights Agenda and Constitutional Rights Project v Nigeria* (1996: par 54):

This article reflects the fact that freedom of expression is a basic human right, vital to an individual’s personal development, his political consciousness, and participation in the conduct of public affairs in his country.

Zimbabwe was also found to have violated Article 9 of the ACHPR (African Union 1981) by the African Commission in the case of Zimbabwe *Lawyers for Human Rights v. Republic of Zimbabwe* (2004). In this case, the African Commission had to decide on the matter of a permanent resident of Zimbabwe who had published information on the Internet that the state did not appreciate. Zimbabwe’s Ministry of Home Affairs deported the permanent resident from the country, and his article was removed from the website. In paragraph 112 of the judgement, the African Commission held that:

It should be recalled that the victim’s deportation arose from the publication of an article that the Respondent State did not appreciate. The Respondent State resorted to deportation to silence him, despite a court order that he could stay in the country. Admittedly, he is not prevented from expressing himself wherever he was deported to, but vis-à-vis his status in the Respondent State, which is a State Party to the African Charter, his ability to express himself as guaranteed under Article 9 was violated.

In this respect, the African Commission ordered the Zimbabwean Government to take measures to rectify this violation of, among other articles, Article 9 of the ACHPR (African Union 1981).

### The scope of Zimbabwe’s media and political participation

Zimbabwe has domesticated the right to access information through Sections 61 and 62 of the Constitution. The dual nature of the media’s right to both access and disseminate information is addressed in Section 61 of the Constitution. Section 62(1) guarantees every citizen, including those with disabilities, the right to access information, particularly regarding public accountability. However, Section 62 does not explicitly address how individuals with visual or hearing impairments can exercise this right. Nevertheless, when Section 62 is interpreted in conjunction with Section 6, which recognises sign language as an official language in Zimbabwe, it may offer guidance on how people with hearing impairments can access information. The enforcement of media access rights in Zimbabwe is done through the Zimbabwe Media Commission (Zimbabwe Government 2020), in conjunction with provisions outlined in the Constitution. Section 248 of the Constitution outlines the responsibilities of the Media Commission, which include upholding, promoting, and developing freedom of media, monitoring broadcasting in the public interest and ensuring fairness and diversity of views that broadly represent the Zimbabwean society.

With the implementation of the Constitution, the Media Commission adopted a more liberal stance regarding the issuance of media licenses, facilitating the emergence of multiple media outlets. According to Ndoma and Moyo-Nyede (2023:8), the current media landscape in Zimbabwe encompasses a variety of sources, including print media, radio, television and social media. This is corroborated by Zirima (2020) in Table 1.

**TABLE 1:** Registered and operational media outlets in Zimbabwe.

Status	Newspaper	Other publications	Radio stations	Television stations	Other broadcasting services	Online or digital outlets
Registered	116	95	16	2	3	11
Operational	33	-	16	1	1	-

*Source*: Zirima, P., 2020, *A media landscape study’ Unpacking the ownership in Zimbabwe’s creation and delivery of News content: A report. Media Monitors Friedrich-Ebert-Stifting*, pp. 15, viewed 20 January 2020, from https://library.fes.de/pdf-files/bueros/simbabwe/19304.pdf

Radio maintains its status as the primary media source, recognised for its extensive reach and influence (Ndoma & Moyo-Nyede 2023:1). However, recent advancements in communication technologies have elevated social media’s role, not only because of its extensive reach but also owing to its capacity for audience engagement in the journalistic process (Mathe & Osunkule 2019:1). Nonetheless, challenges to media access remain entrenched, particularly relating to censorship within state-owned media (Smith 2020:388). As noted by Ndoma and Moyo-Nyede (2023:1), although the Constitution enshrines the rights to media freedom and expression, these rights are significantly undermined by the dominance of state-controlled outlets, with *the Herald* newspaper serving as the principal player in print and the Zimbabwe Broadcasting Corporation (ZBC) being the primary television provider. Furthermore, despite constitutional provisions safeguarding media access for PWDs, resource limitations pose substantial barriers to fully realising the right to information access in Zimbabwe (Dziva et al. 2018:3). Access to media is essential for enabling PWDs to participate in all stages of the electoral process being, pre-election, during the voting and post-election and in their advocacy for political rights.

### Pre-elections stage

In the pre-election stages, media has been used in Zimbabwe as a medium where people can campaign or where the electorate is kept informed about the campaign’s progress. Political parties can also leverage the media to advance their campaigns. According to Hove (2021:1), media can be used to campaign for some political parties, and it can also be used to denounce other political parties. However, equal access to media during campaigns for all interested parties is emphasised by Murray (2019:349). As candidates and political parties engage with the electorate, providing manifestos, agendas and post-election plans, the media undoubtedly serves as a crucial campaign platform (The Electoral Knowledge Network 2012).

Additionally, social media can also be utilised by both candidates and the electorate during election campaigns to foster positive engagement and discourse (Bingisai 2024:183). The rise of social media has notably benefitted emerging political entities, such as the Citizen’s Coalition for Change (CCC), particularly during the 2018 elections, facilitating rapid dissemination of their agenda (Mutanda 2024:6). In the same manner, Zanu Pf engaged in social media to disseminate information about its 2018 annual conference agenda (Marima 2019:3). However, social media information is not screened, verified or further researched before it is posted. Rumours, lies and unsubstantiated information can be posted and may result in misinformation, leading to public confusion (Mutanda 2024:9). Additionally, the impact of social media in Zimbabwe can be hampered by governmental interventions, such as Internet shutdowns and surveillance, as experienced from 14 January 2019 to 18 January 2019 (Tshabangu & Salawu 2024:183).

Legal disputes may also arise during the pre-election stage, and the public gets to know about these developments through the media. In this regard, the media played a very important role in the pre-election phase during the 2023 elections, most importantly the update of the legal dispute of *Kasukuwere v Mangwana and others* (2023) (Kasukuwere case). In this case, Mr Kasukuwere had filed his nomination papers to run for presidency during the 2023 harmonised elections. The first respondent learned from social media about Mr Kasukuwere’s candidature and approached the court for his removal from the list of candidates because he had not been in the country in the past 18 months as required by the Constitution. The Kasukuwere case originated from social media discourse, and subsequent updates on its progress were similarly disseminated through media channels. For instance, on 08 July 2023, *The Herald* (2023b) reported on the status of the case following the High Court Judge’s decision to reserve judgement. This interaction between print media and social media is further exemplified by the inclusion of an excerpt from the Twitter account of Mr. Kasukuwere’s election agent within the same article (Herald 2023b).

Television plays a very significant role in the pre-election stage by broadcasting election campaigns. During the 2023 election campaigns, the Zanu Pf political party’s election campaigns were televised mostly on the main news bulletin (Jogee, Matava & Zvemunyika 2023). It is unfortunate that in Zimbabwe when it comes to the broadcast of election campaigns the ruling party enjoys this platform more than the opposition parties. The opposition political parties would, however, benefit from the international broadcast. For example, the Al-Jazeera on 17 July 2023 broadcasted the launch of the Zimbabwe Opposition political party’s election campaign (Mutasa 2023). Resorting to the use of international media or other sources of media that are not the mainstream media of the country is called alternative media by Tshabangu & Salawu (2024). Alternative media helps to provide access to media on political matters, and they offer an opportunity to the Zimbabweans to hear the other side of politics as they are not state censored.

### Voting update

The media also plays a crucial role in supporting democracy, especially during election periods, by acting as a watchdog during voting (Alfandika & Akpojivi 2020:34; The Electoral Knowledge Network 2012). When the electorate is informed about political parties and candidates through the media, they can make informed decisions in choosing their representatives (Alfandika & Akpojivi 2020:34). Additionally, the media can also be instrumental in updating the public about the election results. The Newsday published an update on the 2023 election results (Masau 2023) as evidenced by the following extract:

The early results announced by the Zimbabwe Electoral Commission (Zec) highlight the continued dominance of the ruling Zanu PF party in rural areas, while the Citizens Coalition for Change (CCC) maintains its stronghold in urban centers. The opposition made significant inroads in Matabeleland North, securing victories in Lupane East, Lupane West and Nkayi South, among other constituencies. In Bulawayo, the opposition CCC achieved a clean sweep of all the contested seats, according to early results released by Zec.

### Post-election stage

The public gets to know of the updates on the post-election landscape and reflections through the media. The outcome of the post-election legal dispute of *Chamisa vs Mnangagwa and 24 others* (2018) was publicised through the media (Hofisi 2019). Political participation is an ongoing practice that transcends the boundaries of elections. In the post-election landscape, the media plays a crucial role in holding political officeholders accountable to the electorate, ensuring transparency and fostering informed public discourse about policy decisions and governance practices. In a survey conducted by Ndoma and Moyo-Nyede (2023:1), it was discovered that the necessity for the media in Zimbabwe is to function as a watchdog over executive actions, ensuring accountability and the exposure of corrupt practices. The media also warns the public of the twist of events in the political direction of the country. Notably, recent media reports have shed light on President Mnangagwa’s stated intentions regarding his future beyond the end of his second and final term. The following is an extract from the *Sunday Mail* newspaper (Maphosa 2025):

The push for President Mnangagwa to remain in office beyond the end of his term in 2028 is unstoppable as it is the will of the people, ZANU PF Mashonaland East provincial chairperson Cde Daniel Garwe has said. He made the remarks while addressing multitudes of Zanu Pf members at the party’s inter-district meeting at Hurungwe Primary School in Murehwa yesterday.

*The Herald* corroborated this position in the following extract (Madzimure 2025):

The push for President Mnangagwa to remain in office beyond the end of his term in 2028 is unstoppable as it is the will of the people, ZANU PF Mashonaland East provincial chairperson Cde Danniel Garwe said. He made the remarks while addressing multitudes of Zanu Pf members at the party’s inter-district meeting at Huringwe Primary School in Murehwa yesterday.

Having this kind of information is necessary for political participation as it prepares the public for the events that are to follow. This can also equip the public to respond and post their opinions through other sources of media such as social media.

#### Political rights advocacy

According to Rugoho (2024:84), there is a strong argument for the media to act as a conduit for marginalised communities, playing a pivotal role in alleviating their hardships. The National Disability Policy (Department of Public Service, Labour and Social Welfare, 2021:67) elaborates on the importance of freedom of expression for PWDs and provides that ‘persons with disabilities must be free to express their opinions, to seek, receive and share information and ideas on an equal basis with others and through all forms, of communication of their choice’. The media has the potential to promote the political rights of PWDs by influencing societal attitudes, beliefs and misconceptions about them (Rugoho 2024:84).

## Access to media by people with disabilities

The above section has established that media is key to political participation. What remains to be analysed, which is at the core of the human rights model of disability, is the extent to which people with visual and hearing disabilities can access media so that they can participate fully like other citizens in the politics of the day. The following analysis will evaluate the accessibility of these media sources for people with visual and hearing disabilities.

### Print media

Print media, encompassing newspapers, magazines and specialised publications such as gazettes and parliamentary records, serves as a vital communication medium for political participation that PWDs must access and benefit from. Print media offers greater diversity in ownership and content compared to monopolised television broadcasting (Electoral Knowledge Network 2012). Newspapers substantially cover political issues and thus contribute significantly to the informational landscape. Tables 2 and 3 show coverage of newspapers on political issues (Zirima 2020).

**TABLE 2:** Percentage space dedicated to different news focus areas for national newspapers.

News outlet	Political and governance (%)	Business and economy (%)	Social and legal (%)	Science and health (%)	Crime and violence (%)	Arts (%)
The Herald	17	24	11	16	2	30
Daily News	3	34	20	7	8	27
NewsDay	25	16	10	10	4	35

*Source*: Zirima, P., 2020, *A media landscape study’ Unpacking the ownership in Zimbabwe’s creation and delivery of News content: A report. Media Monitors Friedrich-Ebert-Stifting,* pp. 15, viewed 24 January 2020, from https://library.fes.de/pdf-files/bueros/simbabwe/19304.pdf

**TABLE 3:** Percentage space dedicated to different news focus areas in weekly newspapers.

News outlets	Politics and governance (%)	Business and economy (%)	Social and legal (%)	Science and health (%)	Crime and violence (%)	Arts (%)
Business Times	2	90	0	0	0	7
Business Weekly	0	69	2	16	1	12
The Sunday Mail	7	34	20	6	5	28
Daily News on Sunday	17	23	1	11	4	45
Financial Gazette	10	76	4	3	1	6
The Standard	16	28	13	5	1	6
Zim Independent	14	68	7	5	0	7
Business Connect	33	36	6	8	3	14
The Patriot	19	25	46	0	1	10

*Source*: Zirima, P., 2020, *A media landscape study’ Unpacking the ownership in Zimbabwe’s creation and delivery of News content: A report. Media Monitors Friedrich-Ebert-Stifting*, pp. 15, viewed 24 January 2020, from https://library.fes.de/pdf-files/bueros/simbabwe/19304.pdf

Print media has the advantage of affordability (Electoral Knowledge Network 2012). For example, both the government-owned *Sunday Mail* and the privately owned *Standard* are priced at one United States Dollar (US$1) and its accessibility to PWDs becomes cheap. In the context of Zimbabwe, print media is predominantly available in urban areas, severely limiting access for PWDs, particularly those in rural regions. This disparity is exacerbated by the fact that individuals without disabilities can more easily navigate urban transport systems, whereas people with visual and hearing disabilities may require assistance. Although print media has the potential to improve communication access for people with visual and hearing loss, especially if made available in Braille, current offerings are insufficient. No newspapers provide Braille versions, indicating a failure to adhere to the human rights model of disability, which requires reasonable accommodation for inclusive access to information for all individuals.

### Radio

Radio as a means of communication can be useful during election time. Radio stations worldwide are important, and according to the Electoral Knowledge Network (2012), in 2002, 95% of the world’s population was covered by at least one radio signal. Zimbabwe has five categories of radio stations: national, provincial, commercial, community and campus radios (Ncube 2021:66; Zimfact 2024). The extensive reach of radio broadcasting can offer opportunities for political engagement. However, the circumstances in Zimbabwe present a distinct scenario, where most radio stations are either state-owned or operated by state-affiliated entities (Electoral Knowledge Network 2012). Out of 37 radio stations in Zimbabwe, only eighty are privately owned commercial radio stations (Zimfact 2024). State-owned radios are susceptible to censorship, thereby limiting public access to unrestricted information. Additionally, people with hearing impairments often cannot benefit from the information that is broadcast through radio without hearing aids. The inaccessibility of this media consequently infringes upon the media access rights of people with hearing impairments to engage in political participation. This situation is inconsistent with the human rights model of disability.

### Television

The ZBC maintains a monopoly over terrestrial television in the country (Ncube 2021:65; Zirima 2020:6). While ZBC holds two television licenses, only one station is currently operational. This station has played a significant role in disseminating election updates and informing the public about the political landscape in Zimbabwe through its main news bulletin (ZBC Online News 2023). Television serves an essential function in election campaigns. The ZBC news incorporates sign language in its main newscasts to accommodate people with hearing disabilities. This practice allows people living with hearing impairments to access media and participate politically by receiving crucial updates related to politics. The ZBC’s recognition of the rights of individuals with hearing impairments aligns with the human rights model of disability. However, there exists an inconsistency in the positioning of the sign language interpreter, who is displayed in a small frame at the corner of the screen, while the main newsreader occupies a significantly larger space (Jogee, Matava & Zvemunyika 2023). This arrangement exemplifies the broader societal treatment of people with hearing disabilities. It is important to note that individuals who are blind cannot benefit from the visual component of this media format, resulting in varied effectiveness in reaching PWDs based on the specific nature of their impairments. Additionally, ZBC’s coverage of political news is limited to only 24%, and there is a lack of sign language interpretation for other politically relevant programmes, such as current affairs (Zirima 2020:24).

In addition to terrestrial programming, satellite television enhances media access with its global reach (Electoral Knowledge Network 2012). The role of satellite television in facilitating balanced political reporting is particularly important in countries like Zimbabwe, where media freedom is restricted. For example, detailed reporting on the opposition party’s campaign launch, the CCC, was made accessible to the public through Al Jazeera broadcasts (Mutasa 2023). Nevertheless, despite the diversity offered by international media, they fall short of fully embracing the human rights disability model because of the absence of sign language interpreters for people with hearing disabilities.

Moreover, access to television media, particularly satellite programming, can be prohibitively expensive, thereby limiting the ability of PWDs to benefit from such resources. The costs associated with acquiring a suitable television set and necessary licenses may be unattainable, especially for individuals living below the poverty line. The requirements for satellite programming, which include purchasing satellite equipment and maintaining periodic subscriptions, exacerbate these financial challenges. Therefore, the realisation of a human rights model of disability necessitates a robust financial commitment dedicated to addressing these issues.

### Social media

Mutanda (2024:1) defines social media as a ‘group of internet-based applications that allow the creation and exchange of user-created content’. Internet-based applications, such as blogs, micro-blogs, Facebook, WhatsApp and Twitter, have become instrumental in political campaigning and election-related communication (Electoral Knowledge Network 2012; Mututwa 2019:94). Social media in Zimbabwe is shaping to be a dominating source of media with some traditional sources of media also posting their content on social media platforms like Facebook (Star FM 2023; ZBC Online News 2023). Leveraging social media for political campaigns and election communication aligns with the human rights model of disability by facilitating participation from people with visual and hearing impairments. These platforms are inherently interactive, fostering audience engagement and transcending geographical limitations, and help PWDs overcome their physical limitations to access information (Bingisai 2024:181). They offer time-efficient communication, resist government censorship and are broadly accessible to a wide demographic on mobile devices, while being cost effective (Bingisai 2024:181; Electoral Knowledge Network 2012). The role of social media as a key source of information for traditional journalists is increasingly important (Electoral Knowledge Network 2012; Herald 2023b).

Because social media is presented in print, audio and videos, it is therefore interactive to both people with visual and hearing disabilities. People with visual disabilities can benefit from the use of inscribed gadgets and built-in screen readers (Baumgartner, Rohrbach & Schönhagen 2021:79). People with hearing disabilities can also participate in social media through typed messages and videos. Social media therefore presents new opportunities for PWDs to share their stories and experiences and to participate in the politics of the day. However, the dynamics of social media leave people with hearing disabilities not fully benefiting from social media because sign language interpretations do not back social media videos. People with visual and hearing impairments may also not fully engage with these social media platforms without adequate assistive technology, which may not be affordable to them as they occupy a lower socio-economic status and are more likely to be unemployed (World Health Organization 2011:10). Additionally, challenges such as geographical disparities, economic barriers and inconsistent energy supplies further exacerbate inequitable Internet access (Electoral Knowledge Network 2012). Ultimately, the core purpose of the human rights disability model remains inadequately met through social media.

## Conclusions and recommendations

The importance of political participation for PWDs cannot be overstated. Numerous national, regional and international legal frameworks affirm the entitlement to political engagement for this demographic. Despite the unequivocal existence of this right, PWDs remain conspicuously underrepresented in parliamentary and other decision-making roles. The unique challenges and limitations posed by their impairments often create barriers to accessing media on an equal footing with individuals without disabilities. This article has appreciated the link between the right to political participation and access to media. Where there is no liberal access to media, people cannot effectively participate in the politics of the day. It is therefore concluded that unless access to media for people with visual and hearing disabilities is improved, their political participation cannot be substantially enhanced. The following recommendations are proposed to foster improved media accessibility and political engagement.

### Legal framework

The *Disabled Persons Act* (Zimbabwe Government 1992) requires an amendment to integrate explicit provisions addressing the political rights and media access for individuals with disabilities, in alignment with the principles outlined in the CRPD. Furthermore, the Constitution should establish consistent terminology and definitions when referring to PWDs across all its provisions to ensure cohesion.

### Improve usage of Braille and sign language in the media

The print media must endeavour to enhance accessibility by providing Braille versions of their newspapers, particularly for political news. In the same vein, sign language should take precedence over verbal narration in news television broadcasts, as it is imperative for the content to be visually accessible to people with hearing disabilities. Sign language may also be used in other television programmes, which are politically related.

### Improve access to media technology

Access to social and online media requires the use of modern devices such as smartphones, laptops and Internet connectivity. The Zimbabwean government may facilitate such access for PWDs by increasing their social assistance grants to enable them to acquire such gadgets. Alternatively, the government may provide subsidised gadgets to PWDs.

### Liberal radio and television licences

In Zimbabwe, the absence of private entities in the television media sector is conspicuous, given that the exclusive ownership and control of the two available television licenses lie with state entities. This monopolistic control renders the media susceptible to manipulation to further the government’s agenda. The introduction of private players into this domain is important to foster diversity and expand the scope of political engagement, particularly for PWDs. The licensing of private operators for radio and television broadcasting holds the potential to enhance inclusivity, with provisions for broadcasting in sign language and furnishing print materials in braille, thereby augmenting accessibility.

### Introduction of sign language and Braille in schools and teaching colleges

To facilitate political participation, it is essential to foster interaction between PWDs and those without. Communication barriers between these groups can hinder political engagement. In Zimbabwe, sign languages and Braille are predominantly taught in specialised disability schools, limiting the ability of PWDs to communicate beyond their immediate community. This presents a significant obstacle for PWDs aspiring to hold political office, as they would need to engage with constituents who do not share their communication methods during campaigns, voting and post-election activities. To address this, Zimbabwe should consider implementing initiatives to incorporate sign language and Braille into mainstream education curricula. This integration would not only benefit PWDs but also facilitate the inclusion of those without disabilities into the communication systems used by the disability community. Such measures are crucial for fostering broader political participation among PWDs. This will also enhance the use of sign language and braille in social media.

### Training and awareness

People with disabilities need to acquire media literacy skills through comprehensive training in the use of communication devices and familiarisation with available media gadgets. Furthermore, disseminating information through awareness campaigns can play a pivotal role in equipping them with essential media knowledge. Training can also be extended to media practitioners on communicating with sign language and Braille. This will improve the interaction between journalists and PWDs. Journalists can resultantly improve the visibility of PWDs in the media and enhance their political participation.

## References

[CIT0001] African Union, 1981, *African Charter on Human and People’s Rights*, Author, Africa Union, viewed 12 October 2024, from https://au.int/en/treaties/african-charter-human-and-peoples-rights.

[CIT0002] African Union, 2018, *Protocol to the African Charter on Human and Peoples’ Rights on the Rights of Persons with Disabilities in Africa*, viewed 12 October 2024, from https://au.int/sites/default/files/treaties/36440-treaty-protocol_to_the_achpr_on_the_rights_of_persons_with_disabilities_in_africa_e.pdf.

[CIT0003] Alfandika, L. & Akpojivi, U., 2020, ‘Contestation of ideas: Media activism and media democracy in Zimbabwe’, *African Journalism Studies* 41(2), 33–48. 10.1080/23743670.2020.1801477

[CIT0004] Baumgartner, A., Rohrbach, T. & Schönhagen, P., 2021, ‘If the phone were broken, I’d be screwed: Media use of people with disabilities in the digital era’, *Disability & Society* 38(1), 73–97. 10.1080/09687599.2021.1916884

[CIT0005] Bingisai, K.C., 2024, ‘Social media and political participation of the youth in contemporary Zimbabwe’, *EAS Journal of Humanities and Cultural Studies* 6(5), 180–187. 10.36349/easjhcs.2024.v06i05.006

[CIT0006] *Chamisa v Mnangagwa & 24 Ors (CCZ 42 of 2018) [2018] ZWCC 42 (24 August 2018)*, viewed 04 February 2025, from https://zimlii.org/akn/zw/judgment/zwcc/2018/42/eng@2018-08-24.

[CIT0007] De Beco, G., 2019, ‘The indivisibility of human rights and the convention on the rights of persons with disabilities’, *International and Comparative Law Quarterly* 68(1), 141–160. 10.1017/S0020589318000386

[CIT0008] Degener, T. & Quinn, G., 2002, ‘A survey of international, comparative and regional disability law reform’, in M.L. Breslin & S. Yee (eds.), *Disability Rights Law and Policy International and national Perspectives*, pp. 3–125, Transnational Publishers Inc., New York.

[CIT0009] Department of Public Service, Labour and Social Welfare, 2021, *The national disability policy*, Author, Veritaszim, Harare.

[CIT0010] Dziva, C., Shoko, M. & Zvobgo, E.F., 2018, ‘Implementation of the 2006 Convention on the Rights of Persons with Disabilities in Zimbabwe: A review’, *African Journal of Disability* 7, 1–7. 10.4102/ajod.v7i0.389 PMC6244346 30473995

[CIT0011] Electoral Knowledge Network, 2012, *Media and elections*, viewed 16 May 2024, from https://aceproject.org/ace-en/topics/me/meb/mab02a.

[CIT0012] Herald, 2023a, *11 Candidates vie for Presidency*, viewed 29 January 2029, from https://www.herald.co.zw/11-candidates-vie-for-presidency/.

[CIT0013] Herald, 2023b, *Judgement reserved in Kasukuwere’s presidential race challenge*, viewed 05 February 2025, from https://www.herald.co.zw/judgment-reserved-in-kasukuweres-presidential-race-challenge/.

[CIT0014] Hofisi, D.T., 2019, ‘High Court rules against Nelson Chamisa… The Battle of Populism vs Constitutionalism’, *The Herald*, 11 May, 2019, viewed 07 February 2025, from https://www.herald.co.zw/high-court-rules-against-nelson-chamisa-the-battle-of-populism-vs-constitutionalism/.

[CIT0015] Hove, R., 2021, ‘A critical analysis of the role of the media in political transition: The Zimbabwean case’, Master Dissertation, Dept. of Arts and Culture, University of South Africa, viewed 29 January 2025, from https://uir.unisa.ac.za/server/api/core/bitstreams/c2ec463a-5eb6-4231-a1e7-817fd34bdcd0/content.

[CIT0016] Jogee, N., Matava, M. & Zvemunyika, P., 2023, *Zimbabwe broadcasting cooperation main news*, viewed 29 January 2025, from https://www.facebook.com/zbcnews/videos/1030180894659237.

[CIT0017] *Kasukuwere vs Mangwana and Others*, 2023, viewed 07 February 2025, from https://www.jsc.org.zw/upload/Judgements/Supreme%20Court/Harare/2023/SC%2078%20-%2023%20Saviour%20Kasukuwere%20vs%20Lovedale%20Mangwana.pdf.

[CIT0018] Kołłątaj, W.P., Kołłątaj, B., Cipora, E., Sygit, K. & Karwat, I.D., 2023, ‘Relationship between the components of disability definition and the effectiveness of rehabilitation measures as a process’, *Annals of Agricultural and Environmental Medicine* 30(4), 595–601. 10.26444/aaem/177341 38153059

[CIT0019] Madzimure, J., 2025, ‘Zanu-PF war vets stand firm on conference resolutions’, *The Herald*, 29 January, 2025, viewed 29 January 2025, from https://www.herald.co.zw/zanu-pf-war-vets-stand-firm-on-conference-resolutions/.

[CIT0020] Maphosa, V., 2025, ‘Presidential term extension unstoppable’, *The Sunday Mail*, 25 January, 2025, viewed 01 February 2025, from https://www.sundaymail.co.zw/presidential-term-extension-unstoppable.

[CIT0021] Maphosa, N., Moyo, C.G. & Moyo, B., 2019, ‘Left in the periphery: An analysis of voting rights for persons with disabilities in Zimbabwe’, *African Disability Rights Yearbook* 7, 112–139. 10.29053/2413-7138/2019/v7a6

[CIT0022] Marima, T., 2019, *Social media in Zimbabwe: A toxic tool or a future bridge to peace. Policy Brief No. 51*, viewed 08 February 2025, from https://toda.org/assets/files/resources/policybriefs/t-pb-51_tendai-marima_social-media-inzimbabwe.pdf.

[CIT0023] Masau, P., 2023, ‘Zanu pf dominates rural constituencies, as CCC makes inroads’, *Newsday*, 25 August, 2023, viewed 29 January 2025, from https://www.newsday.co.zw/2023-elections/article/200015799/zanu-pf-dominates-rural-constituencies-as-ccc-makes-inroads.

[CIT0024] Mathe, L. & Osunkunle, O.O., 2019, ‘Readers’ perspective: Examining the influence of political news in Zimbabwe’, *Communitas* 24, 1–15. 10.18820/24150525/Comm.v24.10

[CIT0025] Media Rights Agenda and Constitutional Rights Project v. Nigeria, 1996, viewed 20 January 2025, from http://hrlibrary.umn.edu/africa/comcases/105-93_128-94_130-94_152-96.html.

[CIT0026] Moyo, A., 2019, ‘Zimbabwe constitutional values, national objectives and the declaration of Rights’, in A. Moyo (ed.), *Selected aspects of the 201 Zimbabwean constitution and the declaration of rights*, pp. 33–34, Raoul Wallenberg Institute of Human Rights and Humanitarian Law, Lund.

[CIT0027] Mtetwa, E., 2016, ‘Disability at the crossroads: The culture of exclusion and the 2013 constitution for Zimbabwe’, *Journal of Social Development in Africa* 31(2), 25–48.

[CIT0028] Murray, R., 2019, *The African Charter on human and peoples’ rights: A commentary*, Oxford Commentaries on International Law; online edn., Oxford Academic, viewed 12 October 2024, from https://0-doi-org.oasis.unisa.ac.za/10.1093/law/9780198810582.003.0014.

[CIT0029] Mutanda, D., 2024, ‘Social media and human development in Zimbabwe: Opportunities and challenges’, *Cogent Arts & Humanities* 11(1), 1–13. 10.1080/23311983.2024.2313850

[CIT0030] Mutasa, H., 2023, ‘Zimbabwe election: Opposition launches campaign’, *Al Jazeera*, viewed 29 January 2025 from https://www.google.com/search?q=2023+election+campaigns+for+zimbabwe&rlz=1C1JJTC_enZA1130ZA1131&oq=2023+election+campaigns+for+zimbabwe&gs_lcrp=EgZjaHJvbWUyBggAEEUYOTIHCAEQIRigAdIBCjIxNDE4ajBqMTWoAgiwAgE&sourceid=chrome&ie=UTF-8#fpstate=ive&vld=cid:244f7e66,vid:P8HmqU1gSyw,st:0.

[CIT0031] Mututwa, W.T., 2019, ‘Facebook image-marketing in Zimbabwe’s 2018 election campaigns: Social media and emerging trends in political marketing’, *Journal of African Elections* 18, 93–111. 10.20940/JAE/2019/v18i2a5

[CIT0032] Naipal, S. & Rampersad, N., 2023, ‘A review of visual impairment’, *Africa Vision Eye Health* 77(1), a393, 1–4. 10.4102/aveh.v77i1.393

[CIT0033] Ncube, L., 2021, ‘Experiences of female journalists in Zimbabwean male-dominated newsrooms’, *Communicae* 40(2), 63–77. 10.36615/jcssa.v40i2.1311

[CIT0034] Ndhlovu, E. & Mudzingwa, N., 2022, ‘Disability inclusion and accessibility in Zimbabwe: Sharing views and experiences of blind and partially sighted persons living in the City of Bulawayo’, *Journal of Public Space* 7(2), 269–278. 10.32891/jps.v7i2.1606

[CIT0035] Ndoma, S. & Moyo-Nyede, S., 2023, *Most Zimbabweans want free media as watchdog over government*, Afrobarometer Dispatch No. 660, viewed 20 January 2025, from https://www.afrobarometer.org/wp-content/uploads/2023/06/AD660-Most-Zimbabweans-want-free-media-as-watchdog-over-government-Afrobarometer-27june23.pdf.

[CIT0036] Oluchina, W.A., 2015, ‘The right to political participation for PWDs in Africa’, *African Disability Rights Yearbook* 3, 309–327. 10.17159/2413-7138/2015/v3n1a14

[CIT0037] Pan, E. & Yanis, T.Z.A., 2023, ‘Affirmative policy a necessity for fulfilling the political rights of persons with disabilities’, *Constitutionale* 4(2), 147–158. 10.25041/constitutionale.v4i2.3164

[CIT0038] Peta, C. & Moyo, A., 2019, ‘The Rights of Persons with Disabilities in Zimbabwe’, in A. Moyo (ed.), *Selected aspects of the 201 Zimbabwean constitution and the Declaration of Rights*, pp. 86–126, Raoul Wallenberg Institute of Human Rights and Humanitarian Law.

[CIT0039] Pillay, P., Saruchera, M. & Chivandire, L., 2023, ‘Unlocking potential examining the effectiveness of non-governmental organisations in empowering PWDs in Zimbabwe’, *African Journal of Public Affairs* 14(1), 19–41.

[CIT0040] Rugoho, T., 2020, ‘Disability representation in digital media in Zimbabwe’, in M. Hoechsmann, G. Thesee, & P.R. Carr (eds.), *Education for democracy; Changing frames of media literacy*, pp. 83–94, Brill.

[CIT0041] Sedova, P., 2024, ‘Human rights model of disability’, in G. Bennett & E. Goodall (eds.), *The Palgrave encyclopedia of disability*, pp. 1–9, Macmillan, Cham.

[CIT0042] Skarstad, K., 2024, ‘Critical human rights research,’ *Journal of Human Rights* 23(3), 297–313. 10.1080/14754835.2024.2354173

[CIT0043] Smith, R.K.M., 2020, *International human rights law*, 10th edn., Oxford University Press, Oxford.

[CIT0044] Star FM News, 2023, *Bulawayo*, 07 November, 2023, viewed 29 January 2025, from https://www.facebook.com/StarFmNews/posts/pfbid07oRnof4P1UWQPH19dufa7tTN9fvbZTUo1zFgV1ehE17D8Vxi85YT6e2jYBWDp5Pvl.

[CIT0045] Tshabangu, T. & Salawu, A., 2024, ‘Alternative media, repression and the crisis state: Towards a Political Economy of alternative media in post Mugabe Zimbabwe’, *Journal of Asian and African Studies* 59(1), 172–186. 10.1177/00219096221106090

[CIT0046] United Nations, 2006, *Convention on the rights of persons with disabilities*, viewed 20 September 2024, from https://www.ohchr.org/en/instruments-mechanisms/instruments/convention-rights-persons-disabilities. 10.1515/9783110208856.203 18348362

[CIT0047] United Nations Committee on the Rights of Persons with Disabilities, 2018, *General comment No. 6 (2018) on equality and non-discrimination*, United Nations Digital Library, viewed 29 January 2025, from https://digitallibrary.un.org/record/1626976?ln=en&v=pd.

[CIT0048] United Nations International Children’s Emergency Fund (UNICEF) Zimbabwe, n.d., *Disabilities*, viewed 20 October 2024, from https://www.unicef.org/Zimbabwe/disabilities.

[CIT0049] Virendrakumar, B., Jolley, E., Badu, E., Murphy, R. & Schmidt, E., 2017, *Disability-inclusive elections in Africa: A qualitative systematic review*, Sightsavers, viewed 24 January 2025, from https://www.sightsavers.org/wp-content/uploads/2018/04/Disability-inclusive-elections-in-Africa-a-qualitative-systematic-review.pdf.

[CIT0050] Witsken, D., 2011, ‘Deaf/hearing impairment’, in J.S. Kreutzer, J. DeLuca & B. Caplan (eds.), *Encyclopedia of clinical neuropsychology*, pp. 7774–777, Springer, New York, NY.

[CIT0051] World Health Organization, 2011, *World report on disability*, viewed 04 February 2025, from https://www.who.int/teams/noncommunicable-diseases/sensory-functions-disability-and-rehabilitation/world-report-on-disability.

[CIT0052] World Health Organization, 2024, *Deafness and hearing loss*, viewed 01 February 2025, from https://www.who.int/news-room/fact-sheets/detail/deafness-and-hearing-loss.

[CIT0053] ZBC News Online, 2023, *Breaking news 2023 general election updates*, 24 August 2023, viewed 29 January 2025, from https://www.facebook.com/watch/live/?ref=watch_permalink&v=833003598336890.

[CIT0054] Zimbabwe Election Support Network 2018, *Report on the 30 July 2018 Harmonised Election*, viewed 20 January 2025, from, https://www.veritaszim.net/sites/veritas_d/files/ZESN-Report-on-the-30-July-2018-Harmonised-Election.pdf.

[CIT0055] Zimbabwe Electoral Commission, 2020, *Post-election gender survey report*, viewed 21 September 2024, from https://zimbabwe.ec-undp-electoralassistance.org/wp-content/uploads/sites/33/2020/12/POST-ELECTION-GENDER-SURVEY-REPORT.pdf.

[CIT0056] Zimbabwe Government, 1992, *Disabled Persons Act*, Veritaszim, Harare.

[CIT0057] Zimbabwe Government, 2013, *Constitution of Zimbabwe Amendment (No. 20) Act*, Author Fidelity Printers and Refiners, Harare.

[CIT0058] Zimbabwe Government, 2020, *Media commission act*, Author Veritaszim, Harare.

[CIT0059] *Zimbabwe Lawyers For Human Rights and Another v Republic of Zimbabwe*, 2009, viewed 07 February 2025, from https://africanlii.org/akn/aa-au/judgment/achpr/2009/98/eng@2009-04-03.

[CIT0060] Zimbabwe Situation, 2015, *Zanu Pf worsened my disability*, Mangoma, 03 July, 2015, viewed 20 January 2025 from https://www.zimbabwesituation.com/news/zimsit-m-zanu-pf-worsened-my-disability-mangoma/.

[CIT0061] Zimfact, 2024, *Factsheet: What is the state of radio broadcasting in Zimbabwe?*, viewed 14 October 2024, from https://zimfact.org/factsheet-what-is-the-state-of-radio-broadcasting-in-zimbabwe/.

[CIT0062] Zirima, P., 2020, *A media landscape study’ Unpacking the ownership in Zimbabwe’s creation and delivery of News content: A report. Media Monitors Friedrich-Ebert-Stifting*, pp. 15, viewed 24 January 2020, from https://library.fes.de/pdf-files/bueros/simbabwe/19304.pdf

